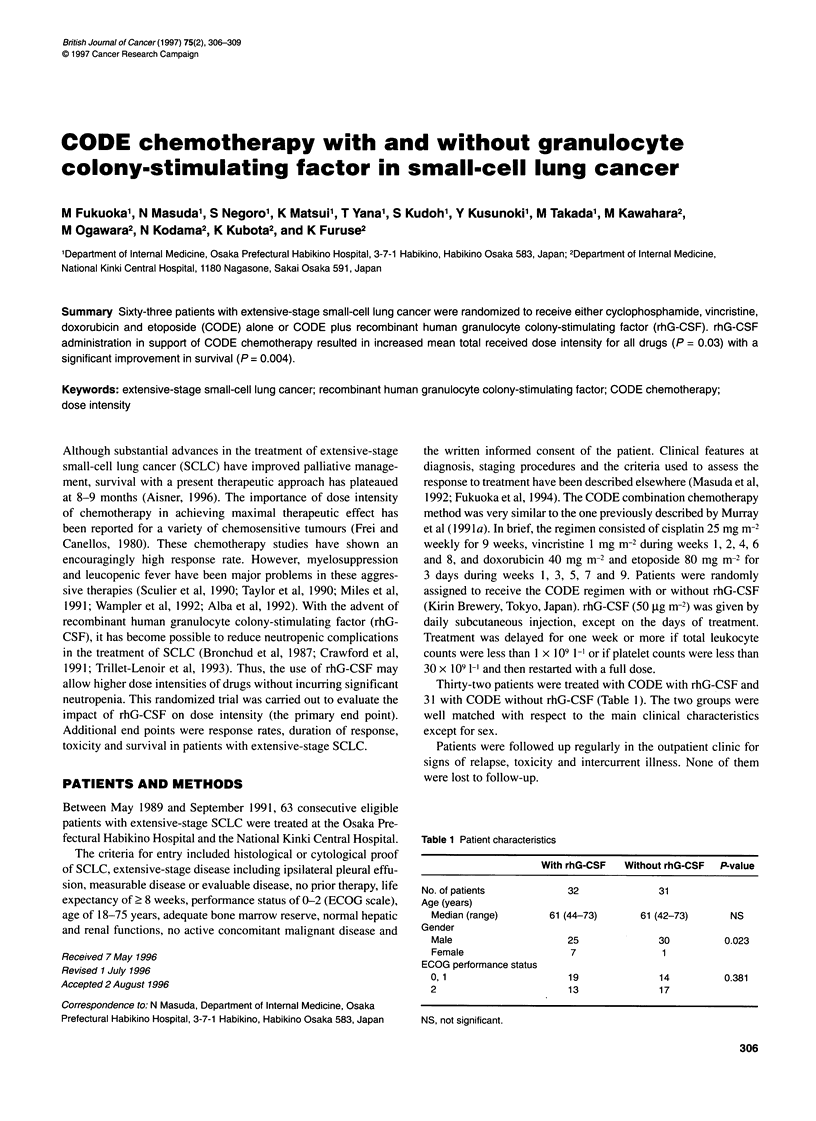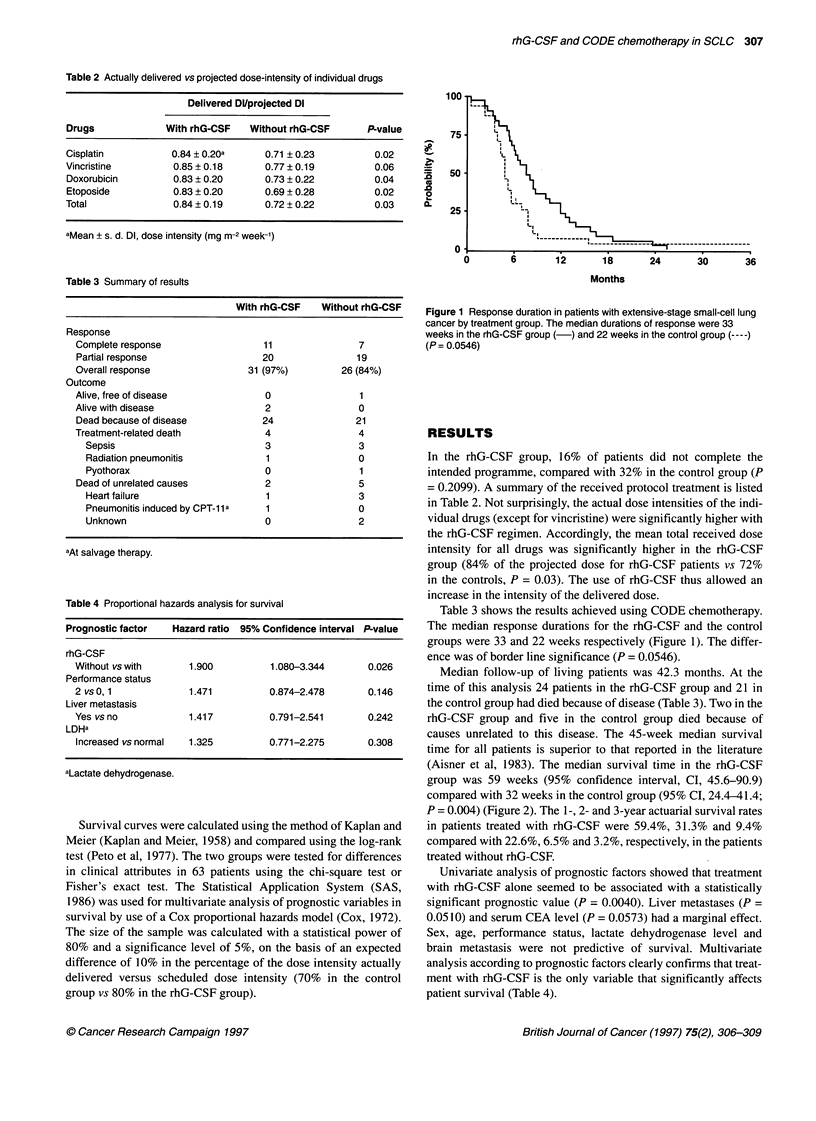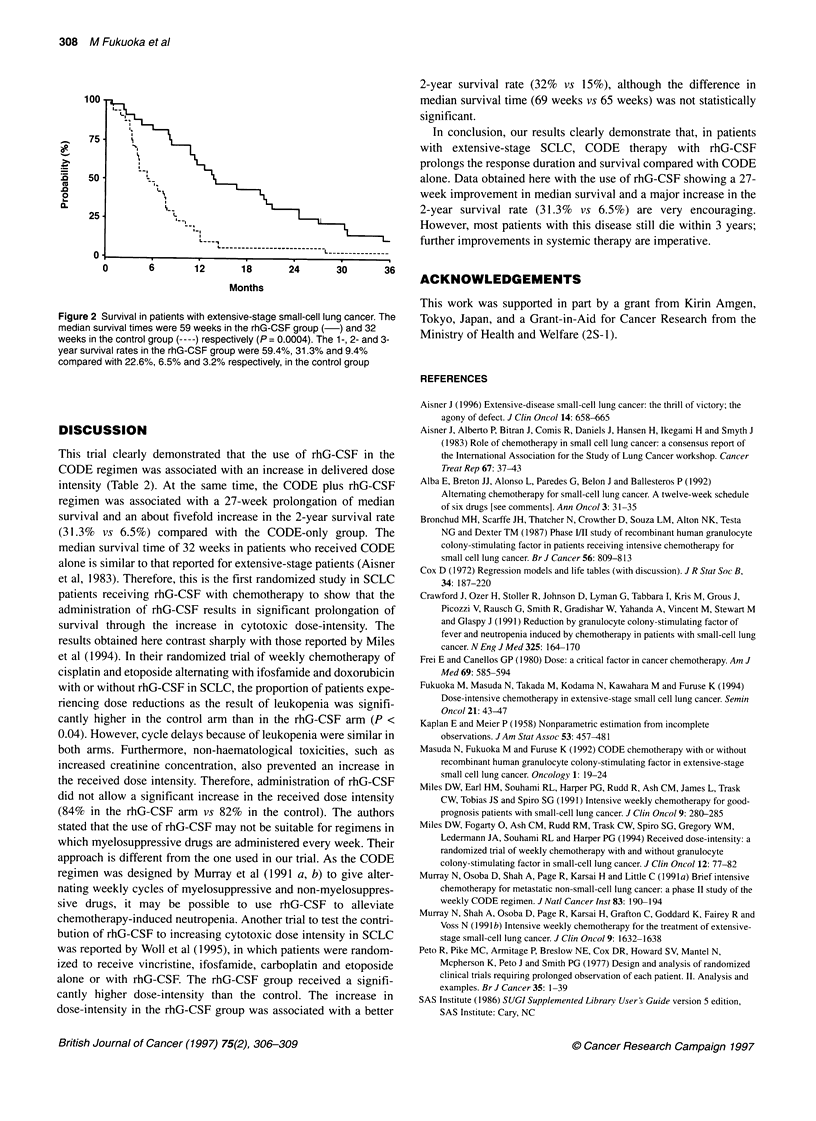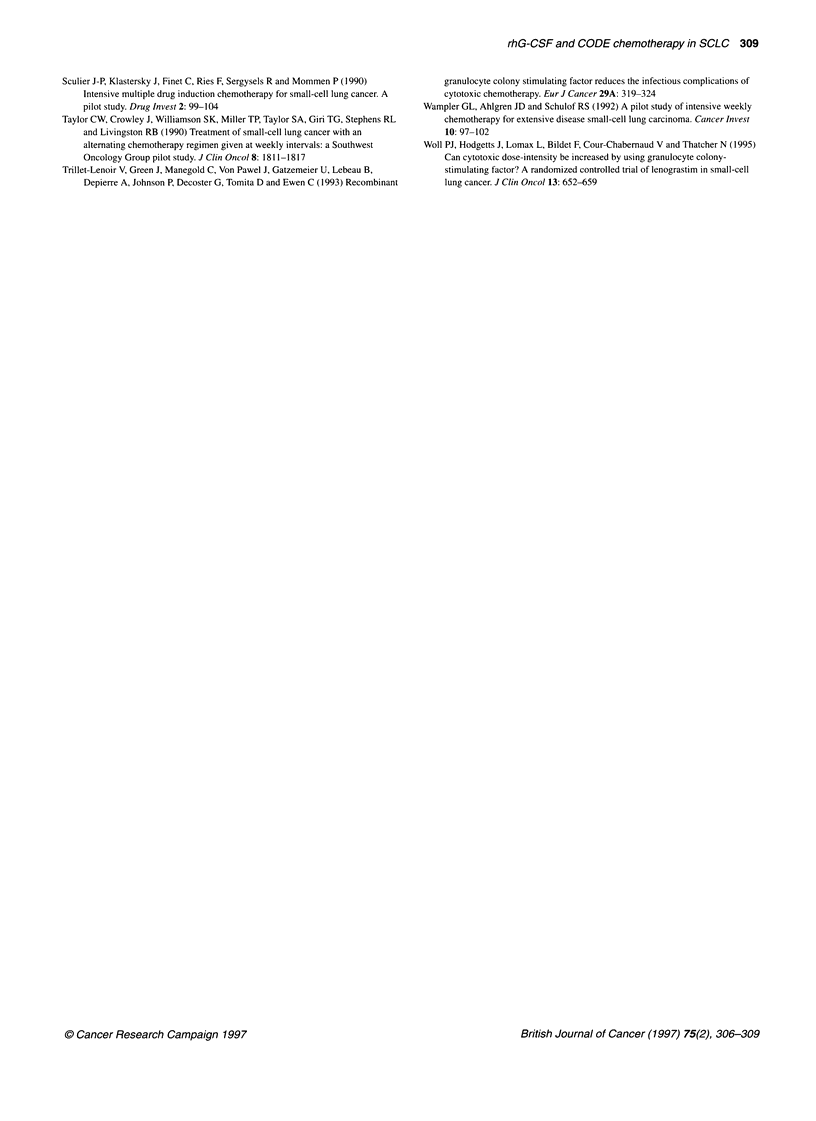# CODE chemotherapy with and without granulocyte colony-stimulating factor in small-cell lung cancer.

**DOI:** 10.1038/bjc.1997.50

**Published:** 1997

**Authors:** M. Fukuoka, N. Masuda, S. Negoro, K. Matsui, T. Yana, S. Kudoh, Y. Kusunoki, M. Takada, M. Kawahara, M. Ogawara, N. Kodama, K. Kubota, K. Furuse

**Affiliations:** Department of Internal Medicine, Osaka Prefectural Habikino Hospital, Japan.

## Abstract

Sixty-three patients with extensive-stage small-cell lung cancer were randomized to receive either cyclophosphamide, vincristine, doxorubicin and etoposide (CODE) alone or CODE plus recombinant human granulocyte colony-stimulating factor (rhG-CSF). rhG-CSF administration in support of CODE chemotherapy resulted in increased mean total received dose intensity for all drugs (P = 0.03) with a significant improvement in survival (P = 0.004).


British Journal of Cancer (1997) 75(2), 306–309
© 1997 Cancer Research Campaign

### CODE chemotherapy with and without granulocyte colony-stimulating factor in small-cell lung cancer

**M Fukuoka[1], N Masuda[1], S Negoro[1], K Matsui[1], T Yana[1], S Kudoh[1], Y Kusunoki[1], M Takada[1], M Kawahara[2], M Ogawara[2], N Kodama[2], K Kubota[2], and K Furuse[2]**

[1]Department of Internal Medicine, Osaka Prefectural Habikino Hospital, 3-7-1 Habikino, Habikino Osaka 583, Japan; [2]Department of Internal Medicine, National Kinki Central Hospital, 1180 Nagasone, Sakai Osaka 591, Japan

**Summary** Sixty-three patients with extensive-stage small-cell lung cancer were randomized to receive either cyclophosphamide, vincristine, doxorubicin and etoposide (CODE) alone or CODE plus recombinant human granulocyte colony-stimulating factor (rhG-CSF). rhG-CSF administration in support of CODE chemotherapy resulted in increased mean total received dose intensity for all drugs ($P$ = 0.03) with a significant improvement in survival ($P$ = 0.004).

**Keywords:** extensive-stage small-cell lung cancer; recombinant human granulocyte colony-stimulating factor; CODE chemotherapy; dose intensity

Although substantial advances in the treatment of extensive-stage small-cell lung cancer (SCLC) have improved palliative management, survival with a present therapeutic approach has plateaued at 8–9 months (Aisner, 1996). The importance of dose intensity of chemotherapy in achieving maximal therapeutic effect has been reported for a variety of chemosensitive tumours (Frei and Canellos, 1980). These chemotherapy studies have shown an encouragingly high response rate. However, myelosuppression and leucopenic fever have been major problems in these aggressive therapies (Sculier et al, 1990; Taylor et al, 1990; Miles et al, 1991; Wampler et al, 1992; Alba et al, 1992). With the advent of recombinant human granulocyte colony-stimulating factor (rhG-CSF), it has become possible to reduce neutropenic complications in the treatment of SCLC (Bronchud et al, 1987; Crawford et al, 1991; Trillet-Lenoir et al, 1993). Thus, the use of rhG-CSF may allow higher dose intensities of drugs without incurring significant neutropenia. This randomized trial was carried out to evaluate the impact of rhG-CSF on dose intensity (the primary end point). Additional end points were response rates, duration of response, toxicity and survival in patients with extensive-stage SCLC.

### PATIENTS AND METHODS

Between May 1989 and September 1991, 63 consecutive eligible patients with extensive-stage SCLC were treated at the Osaka Prefectural Habikino Hospital and the National Kinki Central Hospital.

The criteria for entry included histological or cytological proof of SCLC, extensive-stage disease including ipsilateral pleural effusion, measurable disease or evaluable disease, no prior therapy, life expectancy of ≥ 8 weeks, performance status of 0–2 (ECOG scale), age of 18–75 years, adequate bone marrow reserve, normal hepatic and renal functions, no active concomitant malignant disease and the written informed consent of the patient. Clinical features at diagnosis, staging procedures and the criteria used to assess the response to treatment have been described elsewhere (Masuda et al, 1992; Fukuoka et al, 1994). The CODE combination chemotherapy method was very similar to the one previously described by Murray et al (1991*a*). In brief, the regimen consisted of cisplatin 25 mg $m^{-2}$ weekly for 9 weeks, vincristine 1 mg $m^{-2}$ during weeks 1, 2, 4, 6 and 8, and doxorubicin 40 mg $m^{-2}$ and etoposide 80 mg $m^{-2}$ for 3 days during weeks 1, 3, 5, 7 and 9. Patients were randomly assigned to receive the CODE regimen with or without rhG-CSF (Kirin Brewery, Tokyo, Japan). rhG-CSF (50 μg $m^{-2}$) was given by daily subcutaneous injection, except on the days of treatment. Treatment was delayed for one week or more if total leukocyte counts were less than $1 \times 10^9$ $l^{-1}$ or if platelet counts were less than $30 \times 10^9$ $l^{-1}$ and then restarted with a full dose.

Thirty-two patients were treated with CODE with rhG-CSF and 31 with CODE without rhG-CSF (Table 1). The two groups were well matched with respect to the main clinical characteristics except for sex.

Patients were followed up regularly in the outpatient clinic for signs of relapse, toxicity and intercurrent illness. None of them were lost to follow-up.

**Table 1** Patient characteristics

| | With rhG-CSF | Without rhG-CSF | *P*-value |
|---|---|---|---|
| No. of patients | 32 | 31 | |
| Age (years) | | | |
| Median (range) | 61 (44–73) | 61 (42–73) | NS |
| Gender | | | |
| Male | 25 | 30 | 0.023 |
| Female | 7 | 1 | |
| ECOG performance status | | | |
| 0, 1 | 19 | 14 | 0.381 |
| 2 | 13 | 17 | |

NS, not significant.

*Received 7 May 1996*
*Revised 1 July 1996*
*Accepted 2 August 1996*

*Correspondence to:* N Masuda, Department of Internal Medicine, Osaka Prefectural Habikino Hospital, 3-7-1 Habikino, Habikino Osaka 583, Japan

**Table 2** Actually delivered *vs* projected dose-intensity of individual drugs

| | Delivered DI/projected DI | | |
|---|---|---|---|
| **Drugs** | **With rhG-CSF** | **Without rhG-CSF** | ***P*-value** |
| Cisplatin | 0.84 ± 0.20[a] | 0.71 ± 0.23 | 0.02 |
| Vincristine | 0.85 ± 0.18 | 0.77 ± 0.19 | 0.06 |
| Doxorubicin | 0.83 ± 0.20 | 0.73 ± 0.22 | 0.04 |
| Etoposide | 0.83 ± 0.20 | 0.69 ± 0.28 | 0.02 |
| Total | 0.84 ± 0.19 | 0.72 ± 0.22 | 0.03 |

[a]Mean ± s. d. DI, dose intensity (mg $m^{-2}$ $week^{-1}$)

**Table 3** Summary of results

| | With rhG-CSF | Without rhG-CSF |
|---|---|---|
| Response | | |
| Complete response | 11 | 7 |
| Partial response | 20 | 19 |
| Overall response | 31 (97%) | 26 (84%) |
| Outcome | | |
| Alive, free of disease | 0 | 1 |
| Alive with disease | 2 | 0 |
| Dead because of disease | 24 | 21 |
| Treatment-related death | 4 | 4 |
| Sepsis | 3 | 3 |
| Radiation pneumonitis | 1 | 0 |
| Pyothorax | 0 | 1 |
| Dead of unrelated causes | 2 | 5 |
| Heart failure | 1 | 3 |
| Pneumonitis induced by CPT-11[a] | 1 | 0 |
| Unknown | 0 | 2 |

[a]At salvage therapy.

**Table 4** Proportional hazards analysis for survival

| Prognostic factor | Hazard ratio | 95% Confidence interval | *P*-value |
|---|---|---|---|
| rhG-CSF | | | |
| Without *vs* with | 1.900 | 1.080–3.344 | 0.026 |
| Performance status | | | |
| 2 *vs* 0, 1 | 1.471 | 0.874–2.478 | 0.146 |
| Liver metastasis | | | |
| Yes *vs* no | 1.417 | 0.791–2.541 | 0.242 |
| LDH[a] | | | |
| Increased *vs* normal | 1.325 | 0.771–2.275 | 0.308 |

[a]Lactate dehydrogenase.

Survival curves were calculated using the method of Kaplan and Meier (Kaplan and Meier, 1958) and compared using the log-rank test (Peto et al, 1977). The two groups were tested for differences in clinical attributes in 63 patients using the chi-square test or Fisher's exact test. The Statistical Application System (SAS, 1986) was used for multivariate analysis of prognostic variables in survival by use of a Cox proportional hazards model (Cox, 1972). The size of the sample was calculated with a statistical power of 80% and a significance level of 5%, on the basis of an expected difference of 10% in the percentage of the dose intensity actually delivered versus scheduled dose intensity (70% in the control group *vs* 80% in the rhG-CSF group).

**Figure 1** Response duration in patients with extensive-stage small-cell lung cancer by treatment group. The median durations of response were 33 weeks in the rhG-CSF group (——) and 22 weeks in the control group (----) ($P = 0.0546$)

### RESULTS

In the rhG-CSF group, 16% of patients did not complete the intended programme, compared with 32% in the control group ($P = 0.2099$). A summary of the received protocol treatment is listed in Table 2. Not surprisingly, the actual dose intensities of the individual drugs (except for vincristine) were significantly higher with the rhG-CSF regimen. Accordingly, the mean total received dose intensity for all drugs was significantly higher in the rhG-CSF group (84% of the projected dose for rhG-CSF patients *vs* 72% in the controls, $P = 0.03$). The use of rhG-CSF thus allowed an increase in the intensity of the delivered dose.

Table 3 shows the results achieved using CODE chemotherapy. The median response durations for the rhG-CSF and the control groups were 33 and 22 weeks respectively (Figure 1). The difference was of border line significance ($P = 0.0546$).

Median follow-up of living patients was 42.3 months. At the time of this analysis 24 patients in the rhG-CSF group and 21 in the control group had died because of disease (Table 3). Two in the rhG-CSF group and five in the control group died because of causes unrelated to this disease. The 45-week median survival time for all patients is superior to that reported in the literature (Aisner et al, 1983). The median survival time in the rhG-CSF group was 59 weeks (95% confidence interval, CI, 45.6–90.9) compared with 32 weeks in the control group (95% CI, 24.4–41.4; $P = 0.004$) (Figure 2). The 1-, 2- and 3-year actuarial survival rates in patients treated with rhG-CSF were 59.4%, 31.3% and 9.4% compared with 22.6%, 6.5% and 3.2%, respectively, in the patients treated without rhG-CSF.

Univariate analysis of prognostic factors showed that treatment with rhG-CSF alone seemed to be associated with a statistically significant prognostic value ($P = 0.0040$). Liver metastases ($P = 0.0510$) and serum CEA level ($P = 0.0573$) had a marginal effect. Sex, age, performance status, lactate dehydrogenase level and brain metastasis were not predictive of survival. Multivariate analysis according to prognostic factors clearly confirms that treatment with rhG-CSF is the only variable that significantly affects patient survival (Table 4).

© Cancer Research Campaign 1997 

**Figure 2** Survival in patients with extensive-stage small-cell lung cancer. The median survival times were 59 weeks in the rhG-CSF group (—) and 32 weeks in the control group (----) respectively ($P$ = 0.0004). The 1-, 2- and 3-year survival rates in the rhG-CSF group were 59.4%, 31.3% and 9.4% compared with 22.6%, 6.5% and 3.2% respectively, in the control group

### DISCUSSION

This trial clearly demonstrated that the use of rhG-CSF in the CODE regimen was associated with an increase in delivered dose intensity (Table 2). At the same time, the CODE plus rhG-CSF regimen was associated with a 27-week prolongation of median survival and an about fivefold increase in the 2-year survival rate (31.3% *vs* 6.5%) compared with the CODE-only group. The median survival time of 32 weeks in patients who received CODE alone is similar to that reported for extensive-stage patients (Aisner et al, 1983). Therefore, this is the first randomized study in SCLC patients receiving rhG-CSF with chemotherapy to show that the administration of rhG-CSF results in significant prolongation of survival through the increase in cytotoxic dose-intensity. The results obtained here contrast sharply with those reported by Miles et al (1994). In their randomized trial of weekly chemotherapy of cisplatin and etoposide alternating with ifosfamide and doxorubicin with or without rhG-CSF in SCLC, the proportion of patients experiencing dose reductions as the result of leukopenia was significantly higher in the control arm than in the rhG-CSF arm ($P$ < 0.04). However, cycle delays because of leukopenia were similar in both arms. Furthermore, non-haematological toxicities, such as increased creatinine concentration, also prevented an increase in the received dose intensity. Therefore, administration of rhG-CSF did not allow a significant increase in the received dose intensity (84% in the rhG-CSF arm *vs* 82% in the control). The authors stated that the use of rhG-CSF may not be suitable for regimens in which myelosuppressive drugs are administered every week. Their approach is different from the one used in our trial. As the CODE regimen was designed by Murray et al (1991 *a*, *b*) to give alternating weekly cycles of myelosuppressive and non-myelosuppressive drugs, it may be possible to use rhG-CSF to alleviate chemotherapy-induced neutropenia. Another trial to test the contribution of rhG-CSF to increasing cytotoxic dose intensity in SCLC was reported by Woll et al (1995), in which patients were randomized to receive vincristine, ifosfamide, carboplatin and etoposide alone or with rhG-CSF. The rhG-CSF group received a significantly higher dose-intensity than the control. The increase in dose-intensity in the rhG-CSF group was associated with a better 2-year survival rate (32% *vs* 15%), although the difference in median survival time (69 weeks *vs* 65 weeks) was not statistically significant.

In conclusion, our results clearly demonstrate that, in patients with extensive-stage SCLC, CODE therapy with rhG-CSF prolongs the response duration and survival compared with CODE alone. Data obtained here with the use of rhG-CSF showing a 27-week improvement in median survival and a major increase in the 2-year survival rate (31.3% *vs* 6.5%) are very encouraging. However, most patients with this disease still die within 3 years; further improvements in systemic therapy are imperative.

### ACKNOWLEDGEMENTS

This work was supported in part by a grant from Kirin Amgen, Tokyo, Japan, and a Grant-in-Aid for Cancer Research from the Ministry of Health and Welfare (2S-1).

### REFERENCES

Aisner J (1996) Extensive-disease small-cell lung cancer: the thrill of victory; the agony of defect. *J Clin Oncol* **14**: 658–665

Aisner J, Alberto P, Bitran J, Comis R, Daniels J, Hansen H, Ikegami H and Smyth J (1983) Role of chemotherapy in small cell lung cancer: a consensus report of the International Association for the Study of Lung Cancer workshop. *Cancer Treat Rep* **67**: 37–43

Alba E, Breton JJ, Alonso L, Paredes G, Belon J and Ballesteros P (1992) Alternating chemotherapy for small-cell lung cancer. A twelve-week schedule of six drugs [see comments]. *Ann Oncol* **3**: 31–35

Bronchud MH, Scarffe JH, Thatcher N, Crowther D, Souza LM, Alton NK, Testa NG and Dexter TM (1987) Phase I/II study of recombinant human granulocyte colony-stimulating factor in patients receiving intensive chemotherapy for small cell lung cancer. *Br J Cancer* **56**: 809–813

Cox D (1972) Regression models and life tables (with discussion). *J R Stat Soc B*, **34**: 187–220

Crawford J, Ozer H, Stoller R, Johnson D, Lyman G, Tabbara I, Kris M, Grous J, Picozzi V, Rausch G, Smith R, Gradishar W, Yahanda A, Vincent M, Stewart M and Glaspy J (1991) Reduction by granulocyte colony-stimulating factor of fever and neutropenia induced by chemotherapy in patients with small-cell lung cancer. *N Eng J Med* **325**: 164–170

Frei E and Canellos GP (1980) Dose: a critical factor in cancer chemotherapy. *Am J Med* **69**: 585–594

Fukuoka M, Masuda N, Takada M, Kodama N, Kawahara M and Furuse K (1994) Dose-intensive chemotherapy in extensive-stage small cell lung cancer. *Semin Oncol* **21**: 43–47

Kaplan E and Meier P (1958) Nonparametric estimation from incomplete observations. *J Am Stat Assoc* **53**: 457–481

Masuda N, Fukuoka M and Furuse K (1992) CODE chemotherapy with or without recombinant human granulocyte colony-stimulating factor in extensive-stage small cell lung cancer. *Oncology* **1**: 19–24

Miles DW, Earl HM, Souhami RL, Harper PG, Rudd R, Ash CM, James L, Trask CW, Tobias JS and Spiro SG (1991) Intensive weekly chemotherapy for good-prognosis patients with small-cell lung cancer. *J Clin Oncol* **9**: 280–285

Miles DW, Fogarty O, Ash CM, Rudd RM, Trask CW, Spiro SG, Gregory WM, Ledermann JA, Souhami RL and Harper PG (1994) Received dose-intensity: a randomized trial of weekly chemotherapy with and without granulocyte colony-stimulating factor in small-cell lung cancer. *J Clin Oncol* **12**: 77–82

Murray N, Osoba D, Shah A, Page R, Karsai H and Little C (1991*a*) Brief intensive chemotherapy for metastatic non-small-cell lung cancer: a phase II study of the weekly CODE regimen. *J Natl Cancer Inst* **83**: 190–194

Murray N, Shah A, Osoba D, Page R, Karsai H, Grafton C, Goddard K, Fairey R and Voss N (1991*b*) Intensive weekly chemotherapy for the treatment of extensive-stage small-cell lung cancer. *J Clin Oncol* **9**: 1632–1638

Peto R, Pike MC, Armitage P, Breslow NE, Cox DR, Howard SV, Mantel N, Mcpherson K, Peto J and Smith PG (1977) Design and analysis of randomized clinical trials requiring prolonged observation of each patient. II. Analysis and examples. *Br J Cancer* **35**: 1–39

SAS Institute (1986) *SUGI Supplemented Library User's Guide* version 5 edition, SAS Institute: Cary, NC

© Cancer Research Campaign 1997

Sculier J-P, Klastersky J, Finet C, Ries F, Sergysels R and Mommen P (1990) Intensive multiple drug induction chemotherapy for small-cell lung cancer. A pilot study. *Drug Invest* **2**: 99–104

Taylor CW, Crowley J, Williamson SK, Miller TP, Taylor SA, Giri TG, Stephens RL and Livingston RB (1990) Treatment of small-cell lung cancer with an alternating chemotherapy regimen given at weekly intervals: a Southwest Oncology Group pilot study. *J Clin Oncol* **8**: 1811–1817

Trillet-Lenoir V, Green J, Manegold C, Von Pawel J, Gatzemeier U, Lebeau B, Depierre A, Johnson P, Decoster G, Tomita D and Ewen C (1993) Recombinant granulocyte colony stimulating factor reduces the infectious complications of cytotoxic chemotherapy. *Eur J Cancer* **29A**: 319–324

Wampler GL, Ahlgren JD and Schulof RS (1992) A pilot study of intensive weekly chemotherapy for extensive disease small-cell lung carcinoma. *Cancer Invest* **10**: 97–102

Woll PJ, Hodgetts J, Lomax L, Bildet F, Cour-Chabernaud V and Thatcher N (1995) Can cytotoxic dose-intensity be increased by using granulocyte colony-stimulating factor? A randomized controlled trial of lenograstim in small-cell lung cancer. *J Clin Oncol* **13**: 652–659

© Cancer Research Campaign 1997